# Lysine-specific demethylase 1-mediated demethylation of histone H3 lysine 9 contributes to interleukin 1β-induced microsomal prostaglandin E synthase 1 expression in human osteoarthritic chondrocytes

**DOI:** 10.1186/ar4564

**Published:** 2014-05-16

**Authors:** Fatima Ezzahra El Mansouri, Salwa-Sarah Nebbaki, Mohit Kapoor, Hassan Afif, Johanne Martel-Pelletier, Jean-Pierre Pelletier, Mohamed Benderdour, Hassan Fahmi

**Affiliations:** 1 Department of Medicine, University of Montreal, 2900 Édouard-Montpetit Boulevard, Montreal, QC H3T 1J4, Canada; 2 Osteoarthritis Research Unit, University of Montreal Hospital Research Centre (CRCHUM), 900 rue Saint-Denis, room R11-424, Montreal, QC H2X 0A9, Canada; 3 Research Centre, Sacré-Coeur Hospital, 5400 Gouin Boulevard West, Montreal, QC H4J 1C5, Canada

## Abstract

**Introduction:**

Microsomal prostaglandin E synthase 1 (mPGES-1) catalyzes the terminal step in the biosynthesis of PGE_2_, a critical mediator in the pathophysiology of osteoarthritis (OA). Histone methylation plays an important role in epigenetic gene regulation. In this study, we investigated the roles of histone H3 lysine 9 (H3K9) methylation in interleukin 1β (IL-1β)-induced mPGES-1 expression in human chondrocytes.

**Methods:**

Chondrocytes were stimulated with IL-1β, and the expression of mPGES-1 mRNA was evaluated using real-time RT-PCR. H3K9 methylation and the recruitment of the histone demethylase lysine-specific demethylase 1 (LSD1) to the mPGES-1 promoter were evaluated using chromatin immunoprecipitation assays. The role of LSD1 was further evaluated using the pharmacological inhibitors tranylcypromine and pargyline and small interfering RNA (siRNA)-mediated gene silencing. The LSD1 level in cartilage was determined by RT-PCR and immunohistochemistry.

**Results:**

The induction of mPGES-1 expression by IL-1β correlated with decreased levels of mono- and dimethylated H3K9 at the mPGES-1 promoter. These changes were concomitant with the recruitment of the histone demethylase LSD1. Treatment with tranylcypromine and pargyline, which are potent inhibitors of LSD1, prevented IL-1β-induced H3K9 demethylation at the mPGES-1 promoter and expression of mPGES-1. Consistently, LSD1 gene silencing with siRNA prevented IL-1β-induced H3K9 demethylation and mPGES-1 expression, suggesting that LSD1 mediates IL-1β-induced mPGES-1 expression via H3K9 demethylation. We show that the level of LSD1 was elevated in OA compared to normal cartilage.

**Conclusion:**

These results indicate that H3K9 demethylation by LSD1 contributes to IL-1β-induced mPGES-1 expression and suggest that this pathway could be a potential target for pharmacological intervention in the treatment of OA and possibly other arthritic conditions.

## Introduction

Osteoarthritis (OA) is the most common joint disease and is a leading cause of disability in developed countries and throughout the world [1]. Pathologically, OA is characterized by progressive degeneration of articular cartilage, synovial inflammation and subchondral bone remodeling [2,3]. These processes are thought to be mediated largely through excess production of proinflammatory and catabolic mediators, among which prostaglandin E_2_ (PGE_2_) is considered a critical mediator in the pathophysiology of the disease [2,3]. The beneficial effects of nonsteroidal anti-inflammatory drugs (NSAIDs), the most widely prescribed drugs worldwide, are attributed to inhibition of PGE_2_ production.

PGE_2_ is the most abundant prostaglandin in the skeletal system [4]. Excessive levels of PGE_2_ have been reported in serum and synovial fluid extracted from patients with OA and rheumatoid arthritis (RA) [5]. PGE_2_ contributes to the pathogenesis of OA through several mechanisms, including induction of cartilage proteoglycan degradation [6], upregulation of matrix metalloproteinase (MMP) activity and production [7,8] and promotion of chondrocyte apoptosis [9]. PGE_2_ is also a well-known mediator of pain and neoangiogenesis [10].

The biosynthesis of PGE_2_ requires two enzymes acting sequentially. Cyclooxygenase (COX) enzymes convert arachidonic acid (AA) into PGH_2_, which is in turn isomerized to PGE_2_ by PGE synthase (PGES) enzymes. Two isoforms of the COX enzyme, COX-1 and COX-2, have been identified. COX-1 is expressed in most tissues and is responsible for physiological production of PGs. COX-2, in contrast, is almost undetectable under physiologic conditions, but it is strongly induced in response to proinflammatory and mitogen stimuli [11].

At least three distinct PGES isoforms have been cloned and characterized, including cytosolic prostaglandin E synthase (cPGES), microsomal prostaglandin E synthase 1 (mPGES-1) and mPGES-2 [12]. cPGES, also called the heat shock protein–associated protein p23, is constitutively and ubiquitously expressed with, and functionally coupled with, COX-1, thus promoting immediate production of PGE_2_[13]. In contrast, mPGES-1, which was originally named *membrane-bound glutathione S-transferase-1-like-1* (MGST-L-1), is markedly upregulated by inflammatory or mitogenic stimuli and is functionally coupled with COX-2, thus promoting delayed PGE_2_ production [14]. mPGES-2 is constitutively expressed in various cells and tissues and can be coupled with both COX-1 and COX-2 [15].

We and others have previously shown that expression of mPGES-1, but not of cPGES, is elevated in articular tissues taken from patients with OA [16,17] and patients with RA [18], as well as in the rat adjuvant-induced arthritis model [19], suggesting that aberrant expression of this enzyme might contribute to the pathogenesis of arthritis. Importantly, mPGES-1-deficient mice have been shown to exhibit reduced inflammatory and pain responses and to be protected against experimental arthritis [20-22] and bone loss [23].

The proinflammatory cytokines interleukin 1β (IL-1β) and tumor necrosis factor α (TNF-α) have been shown to induce mPGES-1 expression *in vitro* in several tissue and cell types, including chondrocytes [16,17,24]. However, little is known about the molecular mechanisms underlying the regulation of mPGES-1 expression.

Posttranslational modification of nucleosomal histones, including acetylation, methylation, phosphorylation and sumoylation, play important roles in the regulation of gene transcription through remodeling of chromatin structure [25,26]. To date, histone acetylation and methylation are among the most intensively studied and best characterized modifications of nucleosomal histones. Methylation occurs on both lysine (K) and arginine residues. In histone H3, different lysine residues (K4, K9, K27, K36 and K79) can be methylated. Unlike acetylation, which is generally associated with transcriptional activation, histone lysine methylation is associated with either gene activation or repression, depending on the specific residue modified [27-29].

Methylation of histone H3 lysine 4 (H3K4), H3K36 and H3K79 is generally associated with transcriptionally active genes, whereas methylation of H3K9 and H3K20 is associated with transcription silencing [27-29]. Moreover, lysine methylation can exist in three different states (mono-, di- and trimethylated), which may bring about additional regulatory complexity [27-29].

Lysine methylation is controlled by the opposing activities of lysine methyltransferases (KMTs) and lysine demethylases (KDMs) [27-29]. There are two classes of lysine demethylases: the amine oxidase-related enzymes and the Jumonji (JMJ) C-terminal domain–containing enzymes. Lysine-specific demethylase 1 (LSD1), also known as KDM1, p110b, BHC110 or NPAO, was the first histone demethylase discovered. It belongs to the superfamily of flavin adenine dinucleotide (FAD)–dependent amine oxidases [30]. Researchers in several studies have demonstrated that LSD1 modulates gene expression through demethylation of either H3K4 [31-34] or H3K9 [30,35-38].

In the present study, we demonstrate that the induction of mPGES-1 expression by IL-1β was associated with decreased levels of mono- and dimethylated H3K9 at the mPGES-1 promoter. These changes correlated with the recruitment of the histone demethylase LSD1. Both pharmacological inhibition of LSD1 and small interfering RNA (siRNA) knockdown prevented IL-1β-induced H3K9 demethylation at the mPGES-1 promoter as well as concomitant mPGES-1 protein expression. Furthermore, we show that the level of LSD1 expression was elevated in OA cartilage. These data suggest that modulation of LSD1 in the joint may have therapeutic potential in the treatment of OA and possibly in other conditions associated with increased mPGES-1 expression and PGE_2_ production.

## Methods

### Ethical approval

The Clinical Research Ethics Committee of Notre Dame Hospital approved the study protocol and the use of human articular tissues. Informed consent was obtained from each donor or from a family member.

### Reagents and antibodies

Recombinant human (rh) IL-1β was obtained from Genzyme (Cambridge, MA, USA). Aprotinin, leupeptin, pepstatin, phenylmethylsulfonyl fluoride (PMSF), sodium orthovanadate (Na_3_VO_4_), pargyline and tranylcypromine were purchased from Sigma-Aldrich Canada (Oakville, ON, Canada). Dulbecco’s modified Eagle’s medium (DMEM), penicillin and streptomycin, fetal calf serum (FCS) and TRIzol reagents were supplied by Life Technologies (Burlington, ON, Canada). Abs against mPGES-1 and cPGES-1 were purchased from Cayman Chemical (Ann Arbor, MI, USA). The antibody (Ab) against β-actin was obtained from Santa Cruz Biotechnology (Santa Cruz, CA, USA). Abs against histone H3, mono-, di- and trimethylated H3K9, as well as mono-, di- and trimethylated H3K4, were purchased from EMD Millipore (Billerica, MA, USA). Abs against LSD1/KDM1, JMJD1A/JHDM2A/KDM3A, KIAA1718/JHDM1D/KDM7A, PHF8/JHDM1F/KDM7B and PHF2/JHDM1E/KDM7C were obtained from Abcam (Cambridge, MA, USA). Polyclonal rabbit anti-mouse immunoglobulin G (IgG) antibody, coupled with horseradish peroxidase (HRP) and polyclonal goat anti-rabbit IgG antibody with HRP, were obtained from Thermo Fisher Scientific (Rockford, IL, USA).

### Specimen selection and chondrocyte culture

Human normal cartilage was obtained at necropsy within 12 hours after death from donors with no history of arthritic disease (*n* = 13, mean age ± SD = 56 ± 14 years). To ensure that only normal tissue was used, cartilage specimens were thoroughly examined both macroscopically and microscopically, and only those with neither alteration were further processed. Human OA cartilage was obtained from patients undergoing total knee replacement (*n* = 47, mean age ± SD = 67 ± 20 years). All OA patients were diagnosed on the basis of criteria developed by the American College of Rheumatology Diagnostic Subcommittee for OA [39]. At the time of surgery, the patients had symptomatic disease requiring medical treatment in the form of NSAIDs or selective COX-2 inhibitors. Patients who had received any intraarticular injection of steroids were excluded.

For chondrocyte cultures, cartilage from tibial plateaus and femoral condyles was used. For immunohistochemical studies, only cartilage from femoral condyles was used. Chondrocytes were released from cartilage by sequential enzymatic digestion as previously described [40,41]. Cells were seeded at 3.5 × 10^5^ cells/well in 12-well culture plates (Costar, Corning, NY, USA) or at 6 to 7 × 10^5^ cells/well in 6-well culture plates in DMEM supplemented with 10% FCS, and then they were cultivated at 37°C for 48 hours. The cells were washed and incubated for an additional 24 hours in DMEM containing 0.5% FCS before stimulation with IL-1β alone or in the presence of pharmacological inhibitors of LSD1. Only first-passage chondrocytes were used.

### Western blot analysis

Chondrocytes were lysed in ice-cold lysis buffer (0.1% SDS, 0.5% Nonidet P-40; 50 mM Tris-HCl; pH 7.4; 150 mM NaCl; 2 mM ethylenediaminetetraacetic acid; 1 mM PMSF; 10 μg/ml concentrations each of aprotinin, leupeptin and pepstatin; 1 mM Na_3_VO_4_; and 1 mM NaF). Lysates were sonicated on ice, boiled at 95°C for 5 minutes and centrifuged at 12,000 rpm for 15 minutes. The protein concentration of the supernatant was determined using a bicinchoninic acid protein assay (Thermo Fisher Scientific). Twenty micrograms of total cell lysate were subjected to SDS-PAGE and electrotransferred onto a nitrocellulose membrane (Bio-Rad Laboratories, Mississauga, ON, Canada). After blocking the cell lysate in 20 mM Tris-HCl, pH 7.5, containing 150 mM NaCl, 0.1% Tween 20 and 5% (w/v) nonfat dry milk, blots were incubated overnight at 4°C with the primary Ab and washed with a mixture of Tris-buffered saline, pH 7.5, and 0.1% Tween 20). The blots were then incubated with HRP-conjugated secondary Ab (Thermo Fisher Scientific), washed again, incubated with SuperSignal chemiluminescent substrate (Thermo Fisher Scientific) and exposed to KODAK X-OMAT XAR autoradiography film (Eastman Kodak, Rochester, NY, USA).

### RNA extraction and reverse transcriptase polymerase chain reaction

Total RNA from stimulated chondrocytes was isolated using TRIzol reagent (Life Technologies) according to the manufacturer’s instructions. To remove contaminating DNA, isolated RNA was treated with RNase-free DNaseI (Ambion, Austin, TX, USA). The RNA was quantitated using the RiboGreen RNA assay kit (Molecular Probes, Eugene, OR, USA), dissolved in diethylpyrocarbonate-treated H_2_O and stored at −80°C until use. One microgram of total RNA was reverse-transcribed using Moloney murine leukemia virus reverse transcriptase (Fermentas, Burlington, ON, Canada) as detailed in the manufacturer’s guidelines. One-fiftieth of the reverse transcriptase reaction was analyzed by real-time PCR as described below. The following primers were used: mPGES-1: sense 5′-GAAGAAGGCCTTTGCCAAC-3′ and antisense 5′-GGAAGACCAGGAAGTGCATC-3′; MMP-13: sense 5′-TGAAGCAGTGAAGAAGGAC-3′ and antisense 5′-CTGCTTTCTCTTGTAGAATC-3′; and glyceraldehyde 3-phosphate dehydrogenase (GAPDH): sense 5′-CAGAACATCATCCCTGCCTCT-3′ and antisense 5′-GCTTGACAAAGTGGTCGTTGAG-3′.

### Real-time PCR

Real-time PCR analysis was performed in a total volume of 50 μl containing template DNA, 200 nM concentrations of sense and antisense primers, 25 μl of SYBR Green Master Mix (QIAGEN, Mississauga, ON, Canada) and uracil-*N*-glycosylase (0.5 units, UNG; Epicentre Technologies, Madison, WI, USA). After incubation at 50°C for 2 minutes (UNG reaction) and at 95°C for 10 minutes (UNG inactivation and activation of the AmpliTaq Gold enzyme (Life Technologies)), the mixtures were subjected to 40 amplification cycles (15 seconds at 95°C for denaturation and 1 minute for annealing and extension at 60°C). Incorporation of SYBR Green dye into PCR products was monitored in real time using a GeneAmp 5700 Sequence detection system (Applied Biosystems, Foster City, CA, USA) to enable us to determine the threshold cycle (C_T_) at which exponential amplification of the PCR products began. After PCR, dissociation curves were generated with one peak, which indicated the specificity of the amplification. We obtained a C_T_ value from each amplification curve using the software provided by the manufacturer (Applied Biosystems).

Relative mRNA expression in chondrocytes was determined using the ΔΔC_T_ method, as detailed in the manufacturer’s guidelines (Applied Biosystems). A ΔC_T_ value was first calculated by subtracting the C_T_ value for the housekeeping gene *GAPDH* from the C_T_ value for each sample. A ΔΔC_T_ value was then calculated by subtracting the ΔC_T_ value of the control (unstimulated cells) from the ΔC_T_ value of each treatment. Fold changes compared with the control were then calculated using the 2^−ΔΔCT^ method. Each PCR generated only the expected specific amplicon, as shown by the melting-temperature profiles of the final product and by gel electrophoresis of test PCRs. Each PCR was performed in triplicate on two separate occasions for each independent experiment.

### Chromatin immunoprecipitation assay

The chromatin immunoprecipitation (ChIP) experiments were performed according to the ChIP protocol provided by EMD Millipore. The data are expressed as percentages of control (unstimulated cells) or fold changes relative to control conditions (unstimulated cells) calculated using the ΔΔC_T_ method as detailed in the manufacturer’s guidelines and according to previously published methods [42,43]. A ΔC_T_ value was first calculated by subtracting the C_T_ value for the input DNA from the C_T_ value for the immunoprecipitated sample (ChIP analysis). A ΔΔC_T_ value was then calculated by subtracting the ΔC_T_ value of the control from the ΔC_T_ value of each treatment. Fold changes compared with the control (unstimulated cells) were then calculated using the 2^−ΔΔCT^ method. The following primer sequences were used: mPGES-1 promoter: sense 5′-GTTTGAGGATTTGCCTGGAA-3′ and antisense 5′-CTGCTCATCACCAGGCTGT-3′; and MMP-13 promoter: sense 5′-ATTTTGCCAGATGGGTTTTG-3′ and antisense 5′-CTGGGGACTGTTGTCTTTCC-3′. Primers were tested in a conventional PCR using genomic DNA as the template and checked on an agarose gel to ensure that the primer PCRs resulted in a single band of predicted size.

### RNA interference

Specific siRNA for LSD1 and scrambled control siRNA were obtained from Dharmacon (Lafayette, CO, USA). Chondrocytes were seeded into 6-well plates at 6.10^5^ cells/well and incubated for 24 hours. The cells were then transfected with 100 nM siRNA using HiPerFect Transfection Reagent (QIAGEN) according to the manufacturer’s recommendations. The medium was changed 24 hours later, and then the cells were incubated for an additional 24 hours before stimulation with 100 pg/ml IL-1β for 2 or 20 hours.

### Immunohistochemistry

Cartilage specimens were processed for immunohistochemistry as previously described [40]. The specimens were fixed in 4% paraformaldehyde and embedded in paraffin. Sections (5 μm) of paraffin-embedded specimens were deparaffinized in toluene and dehydrated in a graded series of ethanol. The specimens were then preincubated with chondroitinase ABC (0.25 U/ml in phosphate-buffered saline (PBS) solution, pH 8.0) for 60 minutes at 37°C, followed by a 30-minute incubation with Triton X-100 (0.3%) at room temperature. The slides were then washed in PBS, followed by 2% hydrogen peroxide∕methanol, for 15 minutes. They were then incubated for another 60 minutes with 2% normal serum (Vector Laboratories, Burlingame, CA, USA) and overlaid with primary Ab for 18 hours at 4°C in a humidified chamber. The Ab used was rabbit polyclonal anti-human Set1A Ab (Bethyl Laboratories, Montgomery, TX, USA), which was concentrated at 10 μg/ml. Each slide was washed three times in PBS, pH 7.4, and stained using the avidin-biotin complex method (VECTASTAIN ABC Kit; Vector Laboratories). The color was developed with 3,3′-diaminobenzidine (Vector Laboratories) containing hydrogen peroxide. The slides were counterstained with eosin. The specificity of staining was evaluated using preadsorbed Ab (1 hour, 37°C) with a 20-fold molar excess of protein fragment corresponding to amino acids 834 to 852 of human LSD1 (Abcam) and by substituting the primary Ab with nonimmune rabbit IgG (Chemicon International, Temecula, CA, USA), which was used at the same concentration as the primary Ab. The evaluation of positive-staining chondrocytes was performed using our previously published method [40]. For each specimen, six microscopic fields were examined under 40× magnification. The total number of chondrocytes and the number of chondrocytes staining positive were evaluated, and the results are expressed as the percentage of chondrocytes that stained positive (cell score).

### Flavin adenosine dinucleotide quantification

Intracellular FAD was measured using the FAD Assay and Deproteinizing Sample Preparation Kit (BioVision Research Products, Mountain View, CA, USA).

### Statistical analysis

Data are expressed as the mean ± SD. For chondrocyte culture studies, statistical significance was assessed by one-way analysis of variance, followed by the Bonferroni multiple-comparison *post hoc* test. The comparison of LSD1 expression in human and OA cartilage was analyzed using the two-tailed Student’s *t*-test. *P*-values less than 0.05 were considered statistically significant. All statistics were generated using GraphPad Prism software (GraphPad Software, San Diego, CA, USA).

## Results

### IL-1β decreased H3K9 mono- and dimethylation, but not trimethylation, at mPGES-1 promoter

**Figure 1:**
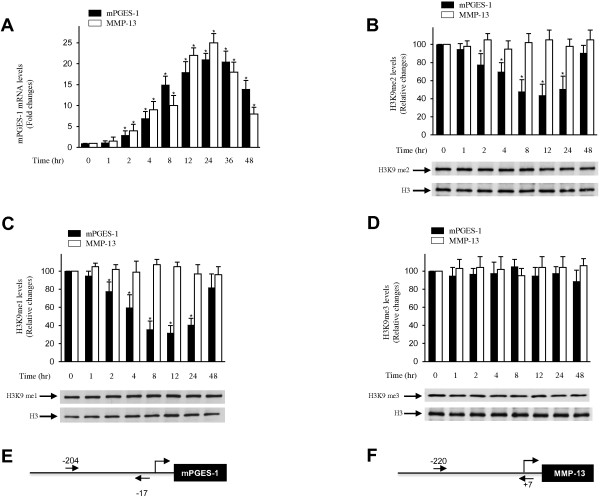
**Effect of interleukin 1β on histone H3 lysine 9 methylation at the microsomal prostaglandin E synthase 1 promoter. (A)** Osteoarthritis (OA) chondrocytes were treated with 100 pg/ml interleukin 1β (IL-1β) for the indicated time periods. Total RNA was isolated, reverse-transcribed into cDNA, and microsomal prostaglandin E synthase 1 (mPGES-1), matrix metalloproteinase 13 (MMP-13) and glyceraldehyde 3-phosphate dehydrogenase mRNAs were quantified using real-time PCR. All experiments were performed in triplicate, and negative controls without template RNA were included in each experiment. The results are expressed as fold changes, assuming 1 as the value of untreated cells, and represent the mean ± SD of four independent experiments using cells from four different OA donors. *P < 0.05 compared with unstimulated cells. **(B)**- through **(D)** Confluent OA chondrocytes were treated with 100 pg/ml IL-1β for the indicated time periods. Chromatin immunoprecipitation (ChIP) assays, coupled with real-time PCR, were performed using antibodies specific to mono- **(B)**, di- **(C)** and trimethylated **(D)** histone H3 lysine 9 (H3K9). me1, Monomethylation; me2, Dimethylation; me3, Trimethylation. The results are expressed as percentages of control values (that is, untreated cells) and are represent the mean ± SD of four independent experiments. For each ChIP assay, the immunoprecipitated DNA was quantitated in triplicate on two separate occasions. *P < 0.05 compared with unstimulated cells. The lower panels show chondrocytes that were treated as indicated. The levels of mono-, di- and trimethylated H3K9 and unmodified H3 were evaluated by immunoblotting. The blots are representative of similar results obtained in four independent experiments in which we used cells from four different OA donors. **(E)** and **(F)** Schematic diagrams of the mPGES-1 and MMP-13 promoters showing the locations of the PCR primers (arrows) used in the ChIP analyses.

First, we examined the effect of IL-1β on mPGES-1 mRNA expression in human OA chondrocytes. The cells were stimulated with IL-1β for various time periods, and the levels of mPGES-1 were determined by real-time RT-PCR. IL-1β-induced changes in mPGES-1 gene expression are expressed as fold changes over control (untreated cells) after normalizing to the internal control GAPDH. As shown in Figure 1A, treatment with IL-1β (100 pg/ml) induced mPGES-1 mRNA expression in a time-dependent manner. mPGES-1 mRNA levels started to increase gradually at 2 hours after stimulation, were significantly increased by 4 hours poststimulation, increased further at 8 hours and peaked at 24 hours. With the longer incubation times, we observed a gradual decline in the mRNA levels starting at 36 hours poststimulation. These results confirmed previously published data showing that IL-1β is a potent inducer of mPGES-1 expression in human OA chondrocytes [16,17,24]. The pattern of *MMP-13* gene expression in response to IL-1β was similar to that of mPGES-1 and hence was used as a control comparator.

In numerous recent studies, researchers have demonstrated that transcriptional activation of a number of genes is associated with changes in the methylation state of H3K9, a critical epigenetic marker for gene silencing [30,35-38]. To determine whether the induction of mPGES-1 by IL-1β was associated with changes in the levels of H3K9 methylation at the mPGES-1 promoter, we performed ChIP assays using specific Abs for mono-, di- or trimethylated H3K9.

Chondrocytes were stimulated with IL-1β for different time periods, and ChIP-enriched DNA was analyzed by real-time PCR using specific primers spanning the transcription start site (+1), the TATA box and several transcription factor binding sites in the proximal regions of the mPGES-1 promoter (bp −259 to +10) and MMP-13 promoter (bp −220 to +7). Control Ig and no Ab were used as controls.As shown in Figures 1B and 1C, treatment with IL-1β decreased the levels of mono- and dimethylated H3K9 at the mPGES-1 promoter in a time-dependent manner. Their levels began to decrease at 2 hours after stimulation with IL-1β, persisted through 12 to 24 hours and then increased at 48 hours. In contrast, the levels of mono- and dimethylated H3K9 at the MMP-13 promoter did not appreciably change under the same conditions (during the treatment) (Figures 1B and 1C), indicating that the observed modifications at the mPGES-1 promoter were specific. There were no significant changes in the levels of trimethylated H3K9 at the mPGES-1 or MMP-13 promoter at any time analyzed (Figure 1D). No immunoprecipitable mPGES-1 promoter DNA was detected with the control Ig or the no-Ab controls (data not shown). The decrease in the levels of mono- and dimethylated H3K9 at the mPGES-1 promoter in response to IL-1β paralleled transcriptional induction of mPGES-1 (Figure 1A), suggesting that diminished levels of mono- and dimethylated H3K9 might play a key role in IL-1β-induced mPGES-1 expression.Next, we investigated the effect of IL-1β on global H3K9 methylation in chondrocytes. Cells were stimulated with IL-1β for various time periods, and the levels of H3K9 methylation were measured by Western blot analysis using specific Abs for mono-, di- and trimethylated H3K9. Figures 1B to 1D demonstrate that the levels of mono-, di- and trimethylated H3K9 were high in untreated chondrocytes and that treatment with IL-1β did not significantly change these levels. These results indicate that the alterations in H3K9 methylation seen in ChIP assays were not due to nonspecific global histone modifications and were specific to the proximal region of the mPGES-1 promoter.

### IL-1β enhanced recruitment of LSD1 to mPGES-1 promoter

**Figure 2:**
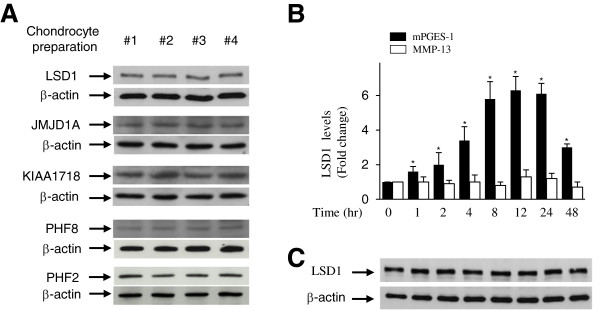
**Effect of interleukin 1β on the recruitment of lysine-specific demethylase 1 to the microsomal prostaglandin E synthase 1. A**, nuclear extracts (20 μg) from four different osteoarthritis (OA) chondrocyte populations obtained from four different donors were studied by Western blot analysis and hybridized to antibodies specific to LSD1/KDM1, JMJD1A/JHDM2A/KDM3A, KIAA1718/JHDM1D/KDM7A, PHF8/JHDM1F/KDM7B and PHF2/JHDM1E/KDM7C. **(B)** Confluent OA chondrocytes were treated with 100 pg/ml interleukin 1β (IL-1β) for the indicated time periods, and chromatin immunoprecipitation (ChIP) assays were performed using a specific antibody against lysine-specific demethylase 1 (LSD1). The results are expressed as fold changes of LSD1 binding to the microsomal prostaglandin E synthase 1 (mPGES-1) or matrix metalloproteinase 13 (MMP-13) promoter relative to untreated cells and represent the mean ± SD of four independent experiments. *P < 0.05 compared with unstimulated cells. **(C)** Confluent OA chondrocytes were treated as described in part **(B)**, and cell lysates were prepared and analyzed for LSD1 protein expression by Western blotting. In the lower panels, the blots were stripped and reprobed with a specific anti-β-actin antibody. The blots are representative of similar results obtained from four independent experiments using cells from four separate donors.

Since the induction of mPGES-1 expression by IL-1β correlated with reduced H3K9 methylation, we hypothesized that IL-1β might mediate this effect by inducing the recruitment of H3K9 demethylases to the mPGES-1 promoter. To test this hypothesis, we first examined whether chondrocytes express the proteins LSD1/KDM1 [30], JMJD1A/JHDM2A/KDM3A [44], KIAA1718/JHDM1D/KDM7A [45], PHF8/JHDM1F/KDM7B [46] and PHF2/JHDM1E/KDM7C [47]. We focused on these proteins because they can demethylate H3K9me1 and H3K9me2, but not H3K9me3. As shown in Figure 2A, Western blot analyses with nuclear extracts from four different chondrocyte populations indicated the presence of the five demethylases in all of the cell populations tested. Hence, we performed ChIP assays to examine whether IL-1β would modulate the recruitment of these demethylases to the mPGES-1 promoter. The results demonstrate that LSD1 was present at the proximal region of the mPGES-1 promoter (Figure 2B) and that treatment with IL-1β enhanced its level in a time-dependent manner. The level started to increase significantly at 2 hours after IL-1β stimulation, reached a maximum at 12 to 24 hours and then decreased by 48 hours. With regard to JMJD1A/JHDM2A/KDM3A, KIAA1718/JHDM1D/KDM7A, PHF8/JHDM1F/KDM7B and PHF2/JHDM1E/KDM7C, their binding signal at the mPGES-1 promoter was undetectable, the C_T_ values were equivalent to that of the nontemplate control (C_T_ ≥38) and IL-1β treatment had no significant effect on their recruitment at the mPGES-1 promoter. No immunoprecipitable mPGES-1 promoter DNA was detected with the control Ig and no-Ab controls (data not shown).Treatment with IL-1β did not affect the levels of LSD1 protein expression (Figure 2C), suggesting that the recruitment of LSD1 to the mPGES-1 promoter seen with the ChIP assays was specific and was not due to increased expression of LSD1 protein.

The pattern of LSD1 levels at the mPGES-1 promoter correlated with decreased H3K9 methylation and is strikingly similar to transcriptional induction of mPGES-1 expression. This strongly suggests that IL-1β-induced mPGES-1 expression involves the recruitment of LSD1 and H3K9 methylation.

### Inhibition of LSD1 activity prevented IL-1β-induced H3K9 demethylation at mPGES-1 promoter and mPGES-1 protein expression

**Figure 3:**
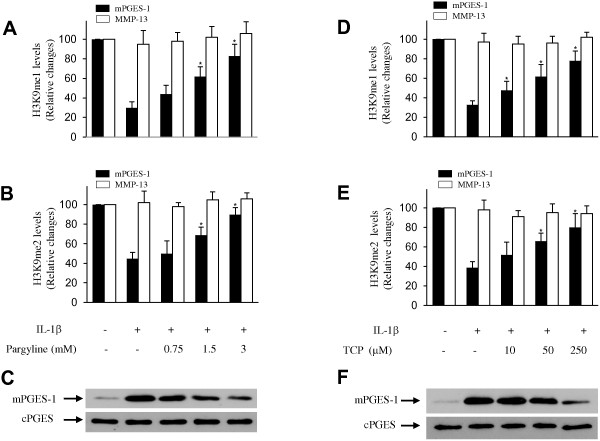
**Effect of pargyline and tranylcypromine on interleukin 1β-induced histone H3 lysine 9 demethylation and microsomal prostaglandin E synthase 1 protein expression.** Osteoarthritis (OA) chondrocytes were pretreated with control vehicle (dimethyl sulfoxide) or increasing concentrations of pargyline **(A)** through **(C)** and tranylcypromine (TCP) **(D)** through **(F)** for 1 hour prior to stimulation with 100 pg/ml interleukin 1β (IL-1β) for 8 hours **(A, B, D and E)** or 24 hours **(C)** and **(F). (A)**, **(B)**, **(D)** and **(E)** Chromatin immunoprecipitation (ChIP) assays, coupled with real-time PCR, were performed using antibodies specific to mono- and dimethylated histone H3 lysine 9 (H3K9). The results are expressed as the percentage of control values (that is, untreated cells) and represent the mean ± SD of four independent experiments. For each ChIP assay, the immunoprecipitated DNA was quantitated in triplicate on two separate occasions. *P < 0.05 compared with IL-1β-treated cells. TCP, tranylcypromine. **(C)** and **(F)** Cell lysates were prepared and analyzed for microsomal prostaglandin E synthase 1 (mPGES-1) protein expression by Western blotting. In the lower panels, the blots were stripped and reprobed with specific anti-β-actin antibody. The blots are representative of similar results obtained in four independent experiments using cells from four separate donors. cPGES, Cytosolic prostaglandin E synthase; me1, Monomethylation; me2, Dimethylation; me3, Trimethylation.

LSD1 demethylates lysine residue through a FAD-dependent reaction [30,48]. This reaction is inhibited by monoamine oxidase inhibitors such as pargyline and tranylcypromine [35,49,50]. Therefore, we investigated their effects on IL-1β-induced H3K9 demethylation at the mPGES-1 promoter and on mPGES-1 protein expression. Chondrocytes were pretreated with increasing concentrations of pargyline or tranylcypromine for 1 hour before stimulation with IL-1β for an additional 8 or 24 hours. The levels of H3K9me1 and H3K9me2 at the mPGES-1 promoter were analyzed using ChIP assays with Abs against mono- and dimethylated H3K9.We found that treatment with either pargyline (Figures 3A and 3B) or tranylcypromine (Figures 3D and 3E) dose-dependently prevented IL-1β-reduced H3K9me1 and H3K9me2 levels, which decreased during transcriptional activation. However, pargyline and tranylcypromine treatment did not change the level of H3K9me3, which was not affected during IL-1β-induced mPGES-1 transcription (data not shown). Accordingly, pretreatment with either pargyline or tranylcypromine dose-dependently suppressed IL-1β-induced mPGES-1 protein expression (Figures 3C and 3F). The inhibition observed was not a result of reduced cell viability, which was confirmed in a 3-(4,5-dimethylthiazol-2-yl)-2,5-diphenyltetrazolium bromide assay (data not shown). These findings strongly suggest that the LSD1 activity contributes to IL-1β-induced H3K9 demethylation at the mPGES-1 promoter as well as to mPGES-1 protein expression.

### LSD1 silencing with siRNA suppressed IL-1β-induced H3K9 demethylation at mPGES-1 promoter and IL-1β-induced mPGES-1 protein expression

**Figure 4:**
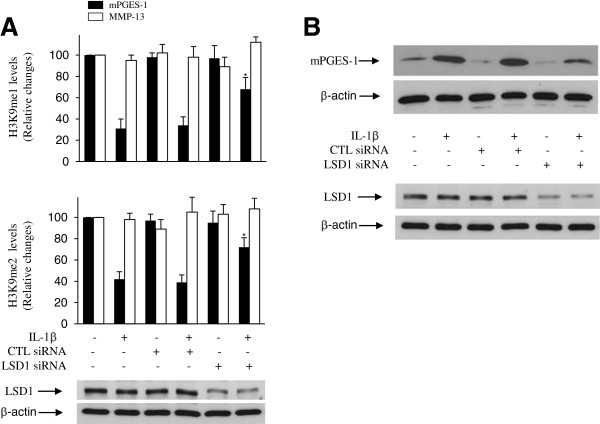
**Effect of lysine-specific demethylase 1 silencing on interleukin 1β–induced histone H3 lysine 9 demethylation at microsomal prostaglandin E synthase 1 promoter.** Osteoarthritis (OA) chondrocytes were transfected with 100 nM control scrambled small interfering RNA (siRNA) or lysine-specific demethylase 1 (LSD1). At 48 hours posttransfection, cells were left untreated or treated with 100 pg/ml interleukin 1β (IL-1β) for 8 hours **(A)** or 24 hours **(B)**. CTL, Control. **(A)** Chromatin immunoprecipitation (ChIP) assays, coupled with real-time PCR, were performed using antibodies specific to mono- and dimethylated histone H3 lysine 9 (H3K9). The results are expressed as percentages of control values (that is, untreated cells), and the data are the mean ± SD of four independent experiments. For each ChIP assay, the immunoprecipitated DNA was quantitated in triplicate on two separate occasions. *P < 0.05 compared with nontransfected cells stimulated with IL-1β. **(B)** Cell lysates were prepared and analyzed for microsomal prostaglandin E synthase 1 (mPGES-1) protein expression by Western blotting. The blots were stripped and reprobed with specific anti-β-actin antibody. The blots are representative of similar results obtained from four independent experiments using cells from four separate donors. Knockdown of LSD1 was confirmed by Western blotting using a specific anti-LSD1 antibody (lower panels).

To further define the role of LSD1, we determined the effect of its silencing by siRNA on IL-1β-induced H3K9 demethylation at the mPGES-1 promoter and on mPGES-1 protein expression. Chondrocytes were transfected with the scrambled control siRNA or siRNA for LSD1, and, after 48 hours of transfection, the cells were either stimulated or not with IL-1β for 8 or 24 hours.As shown in Figure 4, transfection with LSD1 siRNA prevented IL-1β-mediated diminished levels of H3K9me1 and H3K9me2 at the mPGES-1 promoter (Figures 4A and 4B). Furthermore, LSD1 silencing markedly suppressed IL-1β-induced mPGES-1 expression (Figure 4B). In contrast, transfection with scrambled control siRNA had no effect on either H3K9 demethylation or mPGES-1 expression. LSD1 protein levels were reduced by as much as 75% to 80%, confirming gene silencing (Figures 4A and 4B). Together, these data strongly suggest that LSD1 contributed to IL-1β-induced mPGES-1 expression through downregulation of H3K9 mono- and dimethylation.

### Effect of IL-1β on H3K9 methylation, LSD1 recruitment and flavin adenosine dinucleotide levels in normal and osteoarthritis chondrocytes

**Figure 5:**
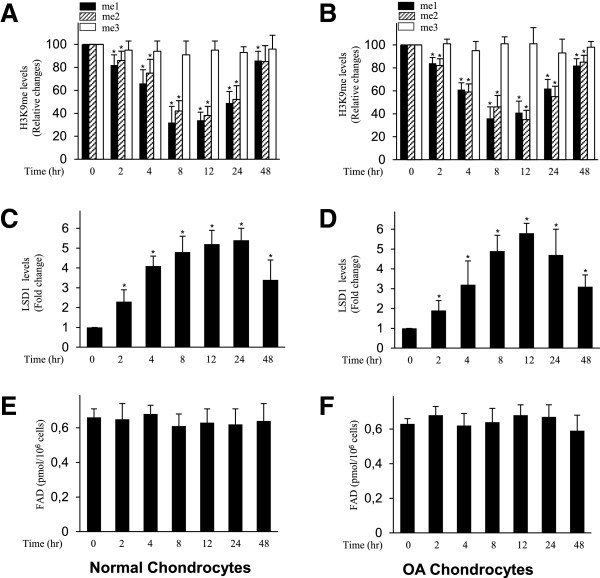
**Effect of interleukin 1 on histone H3 lysine 9 methylation, lysine-specific demethylase 1 recruitment and flavin adenine dinucleotide levels in normal and osteoarthritis chondrocytes.** Normal **(A)** and **(C)** and osteoarthritis (OA) **(B)** and **(D)** chondrocytes were treated with 100 pg/ml interleukin 1β (IL-1β) for the indicated time periods. Chromatin immunoprecipitation (ChIP) assays, coupled with real-time PCR, were performed using antibodies specific to mono-, di- and trimethylated histone H3 lysine 9 (H3K9) **(A)** and **(B)** and lysine-specific demethylase 1 (LSD1) **(C)** and **(D)**. The results are expressed as percentages of control values (that is, untreated cells) or fold changes, and the data are the mean ± SD of three independent experiments using cells from three different donors. *P < 0.05 compared with unstimulated cells. Normal **(E)** and OA **(F)** chondrocytes were treated as indicated, and the levels of flavin adenine dinucleotide (FAD) were determined using a FAD assay kit. The results are expressed in picomolar units per 10^6^ cells, and the data are the mean ± SD of three independent experiments using cells from three different donors. me1, Monomethylation; me2, Dimethylation; me3, Trimethylation.

OA chondrocytes (*n* = 3 donors) and normal chondrocytes (*n* = 3 donors) from age-matched donors were treated with IL-1β for different time periods, and the levels of H3K9 methylation at the mPGES-1 promoter were analyzed by performing ChIP assays using specific Abs for mono-, di- or trimethylated H3K9. We observed a time-dependent decrease in the level of H3K9me2 and H3K9me1 at the mPGES-1 promoter in OA and normal chondrocytes, whereas the level of H3K4me3 remained unchanged (Figures 5A and 5B).Next, we investigated the effect of IL-1β on LSD1 recruitment at the mPGES-1 promoter in normal and OA chondrocytes. As shown in Figure 5C, treatment of normal chondrocytes with IL-1β resulted in LSD1 recruitment at the mPGES-1 promoter, suggesting that, as observed in OA chondrocytes (Figure 5D), the H3K9 demethylase involved in H3K9me1 and H3K9me2 demethylation at the mPGES-1 promoter in normal chondrocytes is LSD1.

LSD1 utilizes FAD as an essential cofactor in catalyzing demethylation of mono- and di-methylated H3K9 [30]. We therefore examined whether IL-1β-induced H3K9 demethylation and LSD1 recruitment to the mPGES-1 promoter were associated with changes in FAD levels. As shown in Figures 5E and 5F, treatment of chondrocytes with IL-1β did not affect the content levels of FAD at any time point. These data indicate that IL-1-induced H3K9 demethylation and LSD1 recruitment in human chondrocytes were not associated with significant changes in FAD levels.

### Effect of IL-1β on histone H3K4 methylation at mPGES-1 promoter

**Figure 6:**
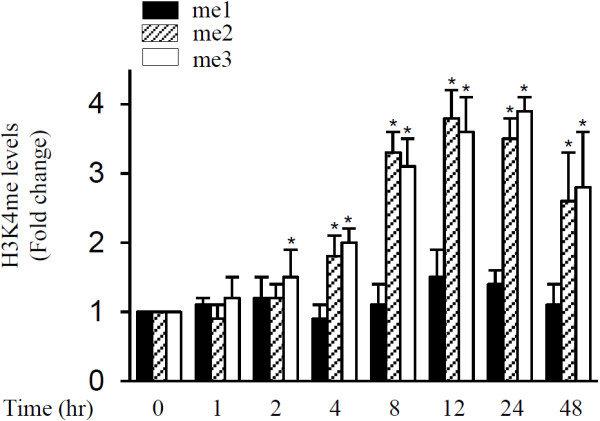
**Effect of interleukin 1β on histone H3 lysine 4 methylation at microsomal prostaglandin E synthase 1 promoter.** Osteoarthritis (OA) chondrocytes were treated with 100 pg/ml interleukin 1β (IL-1β) for the indicated time periods, and chromatin immunoprecipitation (ChIP) assays were performed using antibodies specific to mono-, di- and trimethylated histone H3 lysine 4 (H3K4). The results are expressed as fold changes relative to control (that is unstimulated cells), and the data are the mean ± SD of three independent experiments using cells from four different donors. For each ChIP assay, the immunoprecipitated DNA was quantitated in triplicate on two separate occasions. *P < 0.05 compared with unstimulated cells. me1, Monomethylation; me2, Dimethylation; me3, Trimethylation.

H3K4 methylation is a critical epigenetic marker of transcriptional activation [27-29]. We therefore examined the effect of IL-1β on H3K4 methylation at the mPGES-1 promoter. As shown in Figure 6, treatment with IL-1β enhanced the levels of H3K4 methylation at the mPGES-1 promoter in a time-dependent manner. The levels of di- and trimethylated H3K4 were significantly enhanced at 4 hours after IL-1β stimulation, reached a maximum at 12 hours, persisted through 24 hours and decreased at 48 hours. In contrast, the level of monomethylated H3K4 remained almost unchanged following IL-1β stimulation. The increase in H3K4 di- and trimethylation by IL-1β at the mPGES-1 promoter paralleled the increased transcription of mPGES-1 (Figure 1A), suggesting that, in addition to H3K9 demethylation, H3K4 methylation also contributed to IL-1β-induced mPGES-1 expression.

### LSD1 levels were elevated in osteoarthritis cartilage

**Figure 7:**
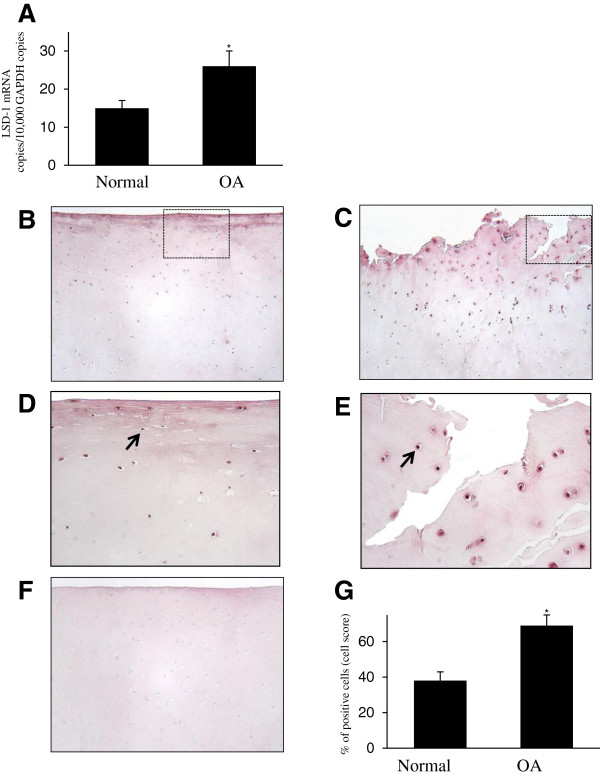
**Expression of lysine-specific demethylase 1 protein in human normal and osteoarthritis cartilage. (A)** RNA was extracted from normal cartilage (*n* = 10) and osteoarthritis (OA) cartilage (*n* = 10), reverse-transcribed into cDNA and processed for real-time PCR. The threshold cycle values were converted to the number of molecules. The data are expressed as copies of the gene’s mRNA detected per 10,000 glyceraldehyde 3-phosphate dehydrogenase (GAPDH) copies. *P < 0.05 versus normal samples. Representative immunostained images of human normal cartilage **(B)** and OA cartilage **(C)** for lysine-specific demethylase 1 (LSD1) protein are shown. **(D)** and **(E)** Higher-magnification views of the areas within the rectangles in **(B)** and **(C)**, respectively. The arrow shows postitive expression of LSD1. **(F)** Cartilage specimens treated with the anti-LSD1 antibody that was preadsorbed with a 20-fold molar excess of the protein fragment corresponding to amino acids 834 to 852 of human LSD1 protein (control for staining specificity). **(G)** Percentage of chondrocytes expressing LSD1 in normal and OA cartilage. The data are the mean ± SD of 10 normal and 10 OA specimens. *P < 0.05 versus normal cartilage.

To investigate the expression of LSD1 *in vivo*, we analyzed its mRNA levels in total cartilage from healthy donors (*n* = 10) and OA donors (n = 10) using real-time quantitative RT-PCR. As shown in Figure 7A, the level of LSD1 mRNA was about 1.7-fold higher in OA cartilage compared with normal cartilage.

(mPGES-1) catalyzes the terminal step in the biosynthesis of PGE_2_, a critical mediator in the pathophysiology of osteoarthritis (OA). Histone methylation plays an important role in epigenetic gene regulation. In this study, we investigated the roles of histone H3 lysine 9 (H3K9).Next, we used immunohistochemistry to analyze the expression level of LSD1 protein. Typical normal and OA cartilage sections immunostained for LSD1 and the corresponding negative control are shown in Figures 7B to 7E. LSD1 expression was seen in normal and OA cartilage in all superficial, middle and deep layers of the articular cartilage, and we observed that the expression levels were relatively high in the superficial and middle zones.

Statistical calculation the cell score revealed that the percentage of cells expressing LSD1 was approximately 1.8-fold higher in OA cartilage (*n* = 10) than in normal cartilage (*n* = 10) (Figure 7G). The specificity of the staining was confirmed using an Ab that had been preadsorbed (1 hour at 37°C) with a 20-fold molar excess of the peptide antigen or nonimmune control IgG (data not shown). Together, these data indicate that the expression level of LSD1 was increased in OA cartilage.

## Discussion

Histone methylation and demethylation play important roles in transcriptional control [27-29]. Histone methylation may positively or negatively regulate gene expression, depending on which residue is modified and how many methyl groups are added. H3K9 methylation usually suppresses transcription, whereas H3K4 methylation generally activates transcription [27-29].

In the present study, we show that IL-1β-induced mPGES-1 expression in human OA chondrocytes correlated with reduced levels of H3K9me1 and H3K9me2 at the mPGES-1 promoter. We identified LSD1 as the responsible demethylase, since inhibition of LSD1 activity or its knockdown prevented IL-1β-induced H3K9 demethylation and mPGES-1 expression. We also demonstrate that LSD1 levels were elevated in the superficial and middle zones of OA cartilage. These data indicate that H3K9 demethylation by LSD1 contributes to IL-1β-induced mPGES-1 expression and suggest that this pathway might be a potential target for modulation of PGE_2_ levels.

Our finding that the induction of mPGES-1 expression by IL-1β was associated with demethylation of H3K9 is consistent with the results of several recent studies in which researchers showed that the transcriptional activation of a number of inducible inflammatory genes correlated with decreased methylation of H3K9 at target promoters. For instance, the induction of IL-12p40, the macrophage-derived chemokine, as well as Epstein-Barr virus–induced molecule 1 ligand chemokine, by lipopolysaccharide (LPS) in dendritic cells was observed to be accompanied by loss of H3K9 methylation at the three gene promoters [51]. Reduced H3K9 methylation was also observed at the IL-1β and TNF-α promoters in LPS-treated THP-1 cells [52,53], at the MMP-9 promoter in phorbol 12-myristate 13-acetate–treated HeLa cells [54] and at the NF-κB-p65 promoter in a model of transient hyperglycemia in bovine aortic endothelial cells [55]. Similarly, H3K9 methylation was reduced upon stimulation of murine vascular smooth muscle cells with TNF-α at the promoters of IL-1β, macrophage colony-stimulating factor 1 and monocyte chemoattractant protein 1 [56]. In line with this finding, and in the context of cancer, transcriptional activation of several genes was associated with decreased H3K9 methylation, including androgen receptor–induced, prostate-specific antigen expression in LNCaP cells [35], as well as estrogen receptor-induced GREB1 expression in MCF7 cells [37]. Loss of H3K9 methylation during varicella zoster virus reactivation from latency has also been reported [38].

Several H3K9me1/2 demethylases have been identified, including LSD1/KDM1 [57], JMJD1A/JHDM2A/KDM3A [44], KIAA1718/JHDM1D/KDM7A [45], PHF8/JHDM1F/KDM7B [46] and PHF2/JHDM1E/KDM7C [47]. We therefore sought to identify which of these demethylases might be involved in the reduction of H3K9me1 and H3K9me2 levels at the mPGES-1 promoter.

Treatment with IL-1β increased the level of LSD1 at the mPGES-1 promoter, but it had no effect on the recruitment of the other demethylases, suggesting that the H3K9 demethylase that is involved in H3K9me1 and H3K9me2 demethylation at the mPGES-1 promoter is LSD1. It is noteworthy that the recruitment of LSD1 at the mPGES-1 promoter coincides with decreased H3K9 mono- and dimethylation and correlates well with the increased transcription of mPGES-1. Taken together, these results strongly suggest that LSD1 recruitment to the mPGES-1 promoter and H3K9 demethylation contribute to IL-1β-induced mPGES-1 expression.

Having established that LSD1 is recruited to the mPGES-1 promoter, we next examined the effect of its pharmacological inhibition or silencing on IL-1β-induced H3K9 demethylation and mPGES-1 expression. The amino oxidase inhibitors tranylcypromine and pargyline, known as potent inhibitors of LSD1 activity, prevented both IL-1β-induced H3K9 demethylation at the mPGES-1 promoter and IL-1β-induced mPGES-1 protein expression. Furthermore, siRNA-mediated LSD1 knockdown suppressed IL-1β-induced H3K9 demethylation and concomitant mPGES-1 protein expression. These results further support the model in which LSD1 contributes to IL-1β-induced mPGES-1 expression through H3K9 demethylation.

Our finding that H3K9 demethylation by LSD1 activates mPGES-1 expression extends similar findings showing transcriptional activation of a number of genes by LSD1. For instance, a genome-wide ChIP assay based on DNA selection and ligation analysis in MCF7 cells treated with 17β-estradiol revealed the presence of LSD1 at 42% of all polymerase II–positive promoters and that 74% of LSD1-positive genes were expressed [37]. Moreover, LSD1 was reported to demethylate H3K9 and to mediate ligand-dependent transcription of both androgen receptor– and estrogen receptor–dependent genes [35,37]. LSD1 was also shown to act as a transcriptional activator during lytic replication of the herpes simplex virus [38], the expression of MMP-9 in retinal endothelial cells [58] and the expression of vascular endothelial growth factor in prostate cancer cells [59].

As stated above, H3K9me1 and H3K9me2 can also be demethylated by JMJD1A/HDM2A/KDM3A [44], KIAA1718/JHDM1D/KDM7A [45], PHF8/JHDM1F/KDM7B [46] and PHF2/JHDM1E/KDM7C [47]. Although we failed to detect the recruitment of these enzymes at the mPGES-1 promoter in our ChIP analysis, we cannot exclude their involvement through binding to other regions of the mPGES-1 promoter, which we did not analyze in the present study. Moreover, our results, which are consistent with key roles of H3K9 demethylation in IL-1β-induced mPGES-1 expression, do not rule out the possibility that changes in the methylation status of other residues might also participate in IL-1β-induced mPGES-1 expression. Indeed, methylation of H3K4, H3K27, H3K36 and H3K79 is known to modulate gene transcription.

Our ChIP findings demonstrate the occupancy of LSD1 at the mPGES-1 promoter in IL-1β-treated cells. However, it is unclear how LSD1 is recruited to the mPGES-1 promoter. One possibility is that LSD1 is recruited to the mPGES-1 promoter by transcriptional regulatory cofactors. Such a mechanism has been reported by Liang *et al*., who demonstrated that the expression of viral immediate early genes in herpes simplex virus and varicella zoster virus involves recruitment of LSD1 by the cellular transcriptional co-activator, host cell factor 1, to viral immediate early promoters [38].

Another possibility is that LSD1 is recruited to the mPGES-1 promoter by transcription factors that play key roles in its transcriptional activation, such as hypoxia-inducible factor 1α (HIF1α) [60] and Krüppel-like factor 5 (KLF5) transcription factor [61]. Indeed, LSD1 has been shown to physically associate with HIF1α in melanoma inhibitory activity human pancreatic carcinoma MIA PaCa-2 cells [62] and with KLF5 in embryonic stem cells [63]. Therefore, it is possible that these transcription factors direct LSD1 to the mPGES-1 promoter. In this context, LSD1 has been shown to be recruited by the androgen receptor and to stimulate transcription through H3K9 demethylation [35]. The transcription factor TLX, an essential neural stem cell regulator, has also been reported to mediate LSD1 recruitment to the promoters of TLX target genes in neural stem cells [64] and Y79 retinoblastoma cells [65].

We also demonstrate that the induction of mPGES-1 by IL-1β was associated with H3K4 methylation. This extends similar previous findings showing H3K4 methylation at the promoters of several inflammatory genes, including inducible nitric oxide synthase and COX-2, in human chondrocytes [40]. The increased level of H3K4 methylation at the mPGES-1 promoter might rely on the ability of LSD1 to anchor other factors at the mPGES-1 promoter rather than on its own enzymatic activity. Indeed, LSD1 is usually found as part of a multiprotein complex with several distinct enzymatic activities, including transcription factors, other demethylases and histone methyltransferases. For instance, Liang *et al*. reported that the activation of α-herpesvirus lytic replication and its reactivation from latency involve H3K9 demethylation and H3K4 trimethylation through recruitment of (1) a multiprotein complex containing LSD1 and (2) the H3K4 methyltransferases mixed lineage leukemia 1 (MLL1) and Set1A [38]. Similarly, Le Douce *et al*. showed that the recruitment of LSD1 at the HIV-1 proximal promoter is associated with both H3K4me3 and H3K9me3 epigenetic markers through corecruitment of LSD1 and the histone methyltransferase hSET1 at the integrated provirus [66]. Wang *et al*. demonstrated that LSD1 associates with the MLL1 complex, which mediates H3K4 trimethylation at the growth hormone promoter during developmental activation [67]. Therefore, it is likely that multiprotein complexes such as these, which contain LSD1 and histone methylases, coordinate H3K4/H3K9 methylation and cooperate to mediate IL-1β-induced mPGES-1 expression.

H3K9 demethylation may mediate IL-1β-induced mPGES-1 expression through several nonexclusive mechanisms. H3K9 demethylation may promote transcriptional activation by enhancing lysine acetylation and allowing better access to DNA for transcription factors and RNA polymerase. Such a mechanism was reported by Escoubet-Lozach *et al*., who showed that LSD1 participates in pomalidomide-induced p21^WAF^ expression in Burkitt’s lymphoma cells by favoring H3K9 acetylation [68]. Similarly, Zhong *et al*. reported that LSD1-mediated MMP-9 expression in diabetes involves increased H3K9 acetylation [58]. H3K9 demethylation can also contribute to IL-1β-induced mPGES-1 expression by preventing DNA methyltransferase recruitment and local DNA methylation, which is often associated with transcriptional silencing. Indeed, H3K9 methylation is required for DNA methylation [69,70]. In addition, H3K9 demethylation can participate in IL-1β-induced mPGES-1 expression by modifying the binding of chromatin factors and/or regulators. In this context, El Gazzar *et al*. demonstrated, in a THP-1 model of endotoxin tolerance, that the loss of H3K9 methylation at the TNF-α promoter induced gene expression by decreasing the level of heterochromatin protein 1 α, which is known for its role in gene silencing [53]. In the present study, we found no evidence of either of these mechanisms. Additional biochemical analyses are clearly warranted to resolve this issue.

We show here that LSD1 expression was higher in OA cartilage than in normal tissue. Interestingly, we and others have previously reported elevated levels of mPGES-1 in OA tissue [16,17,24], suggesting that high expression of LSD1 may be responsible for increased levels of mPGES-1. These data, together with our findings that LSD1 mediates IL-induced mPGES-1 expression in cultured chondrocytes, suggest that elevated levels of LSD1 may be part of the mechanisms responsible for increased mPGES-1 expression in OA cartilage.

## Conclusions

The results of the present study demonstrate that the histone demethylase LSD1 contributes to IL-1β-induced mPGES-1 expression in human chondrocytes through H3K9 demethylation. Our findings thus provide insight into the regulatory mechanisms underlying mPGES-1 expression and may have implications for the design of new anti-OA and anti-inflammatory drugs.

## Abbreviations

AA: Arachidonic acid; ChIP: Chromatin immunoprecipitation; COX: Cyclooxygenase; cPGES: Cytosolic prostaglandin E synthase; C_T_: Threshold cycle; DMEM: Dulbecco’s modified Eagle’s medium; FCS: fetal calf serum; GAPDH: Glyceraldehyde 3-phosphate dehydrogenase; H3K9: Histone H3 lysine 9; HRP: Horseradish peroxidase; Ig: Immunoglobulin; IL: Interleukin; KDM: Lysine demethylase; KMT: Lysine methyltransferase; LPS: Lipopolysaccharide; LSD1: Lysine-specific demethylase; MMP: Matrix metalloproteinase; mPGES-1: Microsomal prostaglandin E synthase 1; mPGES-2: Microsomal prostaglandin E synthase 2; NSAID: Nonsteroidal anti-inflammatory drug; OA: Osteoarthritis; PGE_2_: Prostaglandin E_2_; PMSF: Phenylmethylsulfonyl fluoride; RA: Rheumatoid arthritis; siRNA: Small interfering RNA; TNF-α: tumor necrosis factor α; UNG: Uracil *N*-glycosylase.

## Competing interests

The authors declare that they have no competing interests.

## Authors’ contributions

FEE designed and carried out the cell and real-time RT-PCR and siRNA experiments. SSN contributed to the study design and carried out some of the ChIP and immunoblotting experiments. HA performed some ChIP and immunohistochemistry experiments. MK and MB performed some ChIP experiments and participated in data analysis. JMP and JPP helped by obtaining tissues and participated in immunohistochemistry experiments. HF conceived, designed and coordinated the study; carried out some cell experiments; and drafted the manuscript. All authors contributed to the analysis and interpretation of data and read and approved the final manuscript.
